# Diagnostic Accuracy of Transvaginal Sonography in the Detection of Retained Products of Conception: A Cross-Sectional Study Using Histopathology As the Reference Standard

**DOI:** 10.7759/cureus.108857

**Published:** 2026-05-14

**Authors:** Naseeb Nama, Sana Ambreen, Nadira Kasi, Aisha Tareen, Farzana Bibi

**Affiliations:** 1 Obstetrics and Gynaecology, Sandeman Provincial Hospital, Quetta, PAK; 2 Obstetrics and Gynaecology, University Hospital of North Tees, Stockton-on-Tees, GBR; 3 Obstetrics and Gynaecology, Yoevil Hospital, Yoevil, GBR; 4 Obstetrics and Gynaecology, Bolan Medical College, Quetta, PAK

**Keywords:** diagnostic accuracy, doppler ultrasonography, histopathology, positive predictive value, retained products of conception, sensitivity and specificity, transvaginal sonography

## Abstract

Background

Retained products of conception (RPOC) constitute a clinically significant complication following spontaneous abortion, induced termination, or postpartum delivery, representing a notable contributor to maternal morbidity in resource-limited settings. Timely and accurate diagnosis is paramount to prevent life-threatening sequelae, including hemorrhage, sepsis, and intrauterine adhesive disease. This study aimed to evaluate the diagnostic accuracy of transvaginal sonography (TVS) in detecting RPOC among women of reproductive age, utilizing histopathological examination as the reference gold standard, with stratified analysis across age, gestational age, and symptom duration.

Methodology

This cross-sectional study was conducted at the Department of Obstetrics and Gynaecology, Bolan Medical Complex Hospital (BMCH), Quetta, Pakistan, over a six-month period from October 2022 to April 2023. A total of 205 women aged 15-49 years presenting with clinically suspected RPOC were enrolled through consecutive non-probability sampling. Following thorough clinical evaluation, all participants underwent transvaginal Doppler sonography utilising a 5-7 MHz endovaginal probe. Definitive diagnosis was established through histopathological examination of uterine curettings. Diagnostic performance indices - including sensitivity, specificity, positive predictive value (PPV), negative predictive value (NPV), and overall diagnostic accuracy - were calculated from the 2×2 contingency matrix and stratified across demographic and clinical subgroups. Intergroup comparisons of diagnostic accuracy across age, gestational age, and symptom duration strata were performed using the chi-square test, with a p-value of less than 0.05 considered statistically significant.

Results

Histopathological examination confirmed RPOC positivity in 195 participants (95.12%), while transvaginal sonography yielded positive findings in 168 participants (81.95%). The 2×2 contingency analysis identified 164 true positives, 6 true negatives, 31 false negatives, and 4 false positives. Overall, TVS demonstrated a sensitivity of 84.10%, specificity of 60.00%, PPV of 97.62%, NPV of 16.21%, and diagnostic accuracy of 82.93%. Stratified analysis revealed superior diagnostic performance among younger women aged 15-30 years (accuracy 84.03%), participants with gestational age exceeding 27 weeks (sensitivity 85.52%, specificity 75.00%, accuracy 85.00%), and those presenting within 48 hours of symptom onset (specificity 75.00%, accuracy 84.52%); however, none of the intergroup differences across age (χ² = 0.093, p = 0.7602), gestational age (χ² = 0.274, p = 0.6006), or symptom duration (χ² = 0.312, p = 0.5765) strata attained statistical significance on chi-square testing.

Conclusion

Transvaginal sonography demonstrates clinically acceptable sensitivity and diagnostic accuracy for RPOC detection, with performance optimised at advanced gestational ages and acute symptom presentation. However, the critically low NPV confirms that TVS cannot function as a standalone exclusionary diagnostic tool, necessitating mandatory histopathological corroboration in all sonographically negative yet clinically symptomatic cases within high-prevalence clinical settings.

## Introduction

Retained products of conception (RPOC) represent a clinically significant complication following spontaneous abortion, induced termination of pregnancy, or postpartum delivery, encompassing residual placental or fetal tissue within the uterine cavity. The condition constitutes a major contributor to postpartum and post-abortion morbidity, manifesting as abnormal uterine bleeding, pelvic pain, and fever, with potentially life-threatening sequelae including sepsis, uterine perforation, and Asherman's syndrome if not diagnosed and managed in a timely manner [1,2].

The global burden of RPOC is intrinsically linked to the prevalence of unsafe abortion practices and inadequately managed obstetric care. In developing regions, induced abortions and associated complications remain a substantial cause of maternal morbidity and mortality, with incomplete evacuation of uterine contents representing a frequently encountered clinical scenario [3]. Epidemiological data from Pakistan further highlight the considerable prevalence of unintended pregnancies and induced abortions, underscoring the regional imperative for accurate and accessible diagnostic modalities [4].

Histopathological examination of uterine curettings remains the definitive gold standard for confirming the presence of trophoblastic or chorionic tissue, thereby establishing the diagnosis of RPOC with certainty [5]. However, the invasive nature of surgical curettage mandates prior non-invasive diagnostic evaluation to identify patients who genuinely require intervention. Transvaginal sonography (TVS), particularly with the adjunct of colour Doppler imaging, has emerged as the primary non-invasive investigative tool in this clinical context, offering real-time morphological and vascular characterization of intrauterine contents [6,7].

Despite its widespread clinical utilisation, the diagnostic performance of TVS in detecting RPOC demonstrates variable sensitivity and specificity across published literature, with reported sensitivities ranging from 75% to 93% [8,9]. Such variability is attributable to operator-dependent technique, gestational age at the index event, and heterogeneity in sonographic diagnostic criteria across institutions. A critical appraisal of TVS diagnostic accuracy, stratified by patient demographic and clinical parameters, is therefore essential to delineate its operational utility in clinical practice [10,11].

The present study was designed to evaluate the diagnostic accuracy of transvaginal sonography in detecting RPOC, with histopathology serving as the reference standard, and to examine stratified performance across age, gestational age, and duration of symptomatology.

## Materials and methods

Study design and study setting

The present investigation was structured as a cross-sectional diagnostic accuracy study, a methodological framework well-established for the systematic evaluation of index test performance against a reference gold standard in clinical populations. The study was conducted at the Department of Obstetrics and Gynaecology, Bolan Medical Complex Hospital (BMCH), Quetta, Balochistan, Pakistan.

Study period

The study was conducted over a prospectively defined six-month data collection period, commencing on 13th October 2022 and concluding on 13th April 2023.

Ethics committee approval

Prior to the commencement of any data collection activity, formal ethical approval (Approval No.65/615/2022 dated 25/9/22) was obtained from the Institutional Ethics Committee of Bolan Medical Complex Hospital, Quetta. The study was conducted in full accordance with the Declaration of Helsinki ethical principles governing research involving human participants. All eligible women were provided with a comprehensive explanation of the study's purpose, procedures, potential risks, and anticipated benefits prior to enrolment. Written informed consent was obtained from each participant before any study-related procedure was initiated. Participant confidentiality and data anonymity were maintained throughout all phases of the investigation.

Inclusion criteria

Women fulfilling the following criteria were considered eligible for study enrolment: (i) female sex, aged between 15 and 49 years, encompassing the complete reproductive age spectrum; and (ii) clinical presentation consistent with suspected RPOC, operationally defined as the presence of one or more of the following symptoms - abnormal uterine bleeding, lower abdominal or pelvic pain, and fever - occurring in the context of a recent pregnancy event, including spontaneous abortion, induced termination of pregnancy, or postpartum delivery.

Exclusion criteria

Participants were excluded from the study on the basis of the following predefined criteria: (i) confirmed ongoing intrauterine pregnancy at the time of enrolment; (ii) established diagnosis of coagulopathy or bleeding disorder that would confound clinical symptom interpretation; (iii) history of uterine perforation; (iv) bleeding of perineal or vaginal origin unrelated to RPOC; (v) virginal status with intact hymen, constituting a technical contraindication to safe transvaginal probe insertion, irrespective of reproductive history; (vi) recent pelvic infection that could independently generate sonomorphological findings mimicking RPOC; and (vii) unwillingness to provide written informed consent or to undergo transvaginal sonographic examination.

Operational definitions

The following operational definitions were applied uniformly throughout the study:

Suspected retained Products of Conception (Clinical)

RPOC was clinically suspected when one or more of the following features were present: uterine bleeding assessed on clinical history; pelvic pain with a Visual Analogue Scale (VAS) score exceeding 3; fever defined as axillary temperature exceeding 37.5°C; and uterine tenderness elicited on bimanual clinical examination - all occurring in the context of a recent pregnancy event.

Retained Products of Conception on Transvaginal Sonography

RPOC was diagnosed sonographically when endometrial thickness exceeded 10 mm following dilatation and curettage or spontaneous abortion, assessed using a calibrated 5-7 MHz transvaginal Doppler probe with or without demonstrable colour Doppler vascularity within the intrauterine echogenic mass.

Retained Products of Conception on Histopathology (Reference Standard)

Histopathological confirmation of RPOC was established by the identification of fetal tissues, trophoblasts, or chorionic villi on microscopic examination of uterine curettings, reported by a qualified histopathologist blinded to sonographic findings.

Sample size estimation

Sample size was calculated utilising the Dr. Lin Naing diagnostic accuracy calculator, a validated tool specifically designed for sensitivity and specificity-based sample size estimation in diagnostic accuracy studies. Input parameters were derived from the study by Iqbal et al. (2018), who reported a sensitivity of 75.22% and specificity of 72.50% for ultrasound in detecting RPOC among 210 patients at a tertiary care hospital in Karachi, Pakistan [8]. The estimated disease prevalence of 62.38% was sourced from Karimpour et al. (2010), who documented RPOC prevalence among women presenting with post-abortion complications in a comparable Middle Eastern tertiary care population [12]. Applying a confidence level of 95% (Z = 1.96) and a margin of error of 10%, the Dr. Lin Naing calculator yielded a minimum required sample size of 205 participants, which was adopted as the final target sample size without additional attrition adjustment, given the consecutive enrolment strategy employed at BMCH.

Sampling method

Participants were recruited through a non-probability consecutive sampling technique, wherein all women presenting to the obstetrics and gynaecology outpatient department and emergency unit at BMCH who fulfilled the predefined inclusion criteria were sequentially enrolled until the target sample size was achieved. This approach was selected to minimise selection bias and ensure that the study cohort was representative of the clinical population routinely managed at the study institution, thereby enhancing the external validity and translational applicability of the diagnostic accuracy findings.

Data collection procedure

Following confirmation of eligibility and acquisition of written informed consent, all enrolled participants underwent a standardised, structured data collection protocol. Sociodemographic variables - including age in completed years and categorised into two strata (15-30 years and 31-49 years) - were systematically recorded. Obstetric variables, including gestational age at the index pregnancy event, recorded in weeks and stratified as ≤27 weeks and >27 weeks, were documented through clinical history and corroborated by available antenatal records. Duration of symptomatology from onset to presentation was recorded in hours and categorised as ≤48 hours and >48 hours, reflecting the clinically relevant acute versus sub-acute presentation threshold. Clinical symptoms, including vaginal bleeding, abdominal or pelvic pain, and fever, were assessed through structured clinical examination and patient history. All participants subsequently underwent transvaginal Doppler sonography, performed by a qualified radiologist utilising a calibrated 5-7 MHz endovaginal probe. Sonographic findings were systematically recorded, with RPOC diagnosed sonographically on the basis of an echogenic intrauterine mass with or without demonstrable colour Doppler vascularity. The radiologist performing transvaginal sonography was blinded to the final histopathological results, while the reporting histopathologist was independently blinded to sonographic findings, ensuring bidirectional reference standard independence and minimising partial verification bias. The final definitive diagnosis was established through histopathological examination of uterine curettings obtained via surgical evacuation, with findings reported by a qualified histopathologist blinded to sonographic results, thereby ensuring reference standard independence and minimising incorporation bias.

Data analysis

All collected data were entered and analysed utilising SPSS version 23.0 (IBM Corp., Armonk, NY). Descriptive statistics were computed for all demographic and clinical variables, with continuous variables expressed as mean ± standard deviation and categorical variables presented as frequencies and percentages. Diagnostic performance indices - including sensitivity, specificity, positive predictive value PPV, NPV, and overall diagnostic accuracy - were derived from the 2×2 contingency matrix constructed against histopathological findings as the reference gold standard. Stratified diagnostic accuracy analyses were performed across age, gestational age, and symptom duration subgroups. Intergroup comparisons of diagnostic performance indices across stratification subgroups were performed using the chi-square test for proportions. A p-value of less than 0.05 was considered statistically significant throughout all analyses.

## Results

The baseline demographic and clinical profile of the 205 enrolled participants demonstrated a bimodal age distribution, with the majority comprising younger reproductive-age women: 119 participants (58.00%) were aged between 15 and 30 years, while the remaining 86 participants (42.00%) fell within the 31-49-year stratum, yielding a mean age of 29.00 years with a standard deviation of 5.71 years (Table 1). With respect to gestational age, 125 participants (60.98%) presented at or below 27 weeks of gestation, while 80 participants (39.02%) exceeded this threshold, with a mean gestational age of 20.00 weeks (SD ± 8.83 weeks). Regarding duration of symptomatology, 121 participants (59.02%) reported a symptom duration exceeding 48 hours, suggesting a predominantly sub-acute clinical presentation across the study cohort.

**Table 1:** Baseline Demographic and Clinical Characteristics of Study Participants with Suspected Retained Products of Conception Data presented as n (%) for categorical variables and mean ± SD for continuous variables. SD = standard deviation; POG = period of gestation.

Parameter	Category	Frequency (n)	Percentage (%)
Age (years)	15–30	119	58.00
	31–49	86	42.00
	Total	205	100.00
	Mean ± SD	29.00 ± 5.71 years	-
Gestational age (weeks)	≤27 weeks	125	60.98
	>27 weeks	80	39.02
	Total	205	100.00
	Mean ± SD	20.00 ± 8.83 weeks	-
Duration of symptoms	≤48 hours	84	40.98
	>48 hours	121	59.02
	Total	205	100.00
	Mean ± SD	48.00 ± 10.09 hours	-

Analysis of diagnostic findings across both modalities revealed a notably high prevalence of histopathologically confirmed RPOC within the study cohort, with 195 participants (95.12%) demonstrating positive findings on uterine curettage histopathology, while only 10 participants (4.88%) returned negative results (Table 2). Transvaginal sonography, by contrast, identified RPOC in 168 participants (81.95%), with the remaining 37 participants (18.05%) yielding negative sonographic findings. The discordance between histopathological positivity rate (95.12%) and sonographic positivity rate (81.95%) is clinically noteworthy and underscores the inherent diagnostic limitations of sonography, particularly with respect to false-negative determinations, which carry material implications for patient management decisions.

**Table 2:** Distribution of Histopathological and Sonographic Diagnostic Findings Among Study Participants Data presented as n (%). Histopathological examination served as the reference gold standard. RPOC = retained products of conception; TVS = transvaginal sonography

Diagnostic Modality	Findings	Frequency (n)	Percentage (%)
RPOC on histopathology (gold standard)	Positive	195	95.12
	Negative	10	4.88
	Total	205	100.00
RPOC on transvaginal sonography	Positive	168	81.95
	Negative	37	18.05
	Total	205	100.00

The contingency matrix in Table 3 established 164 true positives and 6 true negatives against 31 false negatives and 4 false positives, demonstrating that transvaginal sonography correctly classified 170 of 205 participants. The 31 false-negative cases are clinically consequential, indicating that approximately one in six histopathologically confirmed RPOC cases escaped sonographic detection within this high-prevalence cohort.

**Table 3:** 2×2 Contingency Matrix of Transvaginal Sonography Against Histopathological Gold Standard for Detection of Retained Products of Conception (n=205) TP = true positive; FP = false positive; FN = false negative; TN = true negative. TVS = transvaginal sonography. Histopathological examination served as the reference gold standard.

Variables	Histopathology Positive	Histopathology Negative	Row Total
TVS positive	164 (TP)	4 (FP)	168
TVS negative	31 (FN)	6 (TN)	37
Column total	195	10	205

Transvaginal sonography demonstrated a sensitivity of 84.10%, specificity of 60.00%, PPV of 97.62%, NPV of 16.21%, and overall diagnostic accuracy of 82.93% (Table 4). The markedly elevated PPV reflects the exceptionally high disease prevalence of 95.12%, while the critically low NPV of 16.21% establishes that a negative TVS result carries insufficient diagnostic certainty to exclude RPOC in this clinical population.

**Table 4:** Derived Diagnostic Performance Indices of Transvaginal Sonography for Retained Products of Conception Against Histopathological Reference Standard (n=205) TP = true positive; FP = false positive; FN = false negative; TN = true negative. All indices derived from the 2×2 contingency matrix presented in Table 3.

Diagnostic Index	Calculated Value
Sensitivity	84.10%
Specificity	60.00%
Positive predictive value	97.62%
Negative predictive value	16.21%
Overall diagnostic accuracy	82.93%

Age-stratified analysis in Table 5 demonstrated comparable sensitivity across both cohorts - 84.96% in women aged 15-30 years versus 84.14% in women aged 31-49 years - with no statistically significant intergroup difference detected (χ² = 0.021, p = 0.8847). Specificity was marginally superior in the younger cohort at 66.67% compared to 50.00% in the older group, though this difference did not achieve statistical significance (χ² = 0.381, p = 0.5370). Overall diagnostic accuracy was 84.03% versus 82.55%, respectively, with no statistically significant intergroup difference (χ² = 0.093, p = 0.7602), indicating that age alone does not significantly influence TVS diagnostic performance for RPOC within this cohort.

**Table 5:** Stratified Diagnostic Accuracy of Transvaginal Sonography with Respect to Age Group with Intergroup Comparison Data presented as absolute values and percentages. Age stratification was performed at the 30-year threshold. P < 0.05 was considered statistically significant.

Diagnostic Index	Age 15-30 Years (n=119)	Age 31-49 Years (n=86)	Chi-square value	p-value
True positive	96	69	-	-
False positive	2	2	-	-
False negative	17	13	-	-
True negative	4	2	-	-
Histopathology positive	113	82	-	-
Histopathology negative	6	4	-	-
Sensitivity	84.96%	84.14%	0.021	0.8847
Specificity	66.67%	50.00%	0.381	0.5370
Positive predictive value	97.95%	97.18%	0.144	0.7044
Negative predictive value	19.04%	13.33%	0.112	0.7376
Diagnostic accuracy	84.03%	82.55%	0.093	0.7602

Gestational age-stratified analysis in Table 6 revealed a clinically meaningful divergence in specificity - 75.00% in the >27-week subgroup versus 50.00% in the ≤27-week subgroup - and in overall diagnostic accuracy (85.00% versus 82.40%); however, neither difference attained statistical significance on chi-square testing (specificity: χ² = 0.686, p = 0.4077; accuracy: χ² = 0.274, p = 0.6006). The consistent trend toward superior performance at advanced gestational ages, despite non-significant p-values, likely reflects the limited statistical power attributable to the small number of true-negative cases in both subgroups, warranting larger-scale confirmatory studies.

**Table 6:** Stratified Diagnostic Accuracy of Transvaginal Sonography with Respect to Gestational Age with Intergroup Comparison Data presented as absolute values and percentages. Gestational age threshold set at 27 weeks per study protocol. P < 0.05 is considered statistically significant.

Diagnostic Index	Gestational Age ≤27 Weeks (n=125)	Gestational Age >27 Weeks (n=80)	Chi-square value	p-value
True positive	100	65	-	-
False positive	3	1	-	-
False negative	19	11	-	-
True negative	3	3	-	-
Histopathology positive	119	76	-	-
Histopathology negative	6	4	-	-
Sensitivity	84.03%	85.52%	0.082	0.7746
Specificity	50.00%	75.00%	0.686	0.4077
Positive predictive value	97.08%	98.48%	0.337	0.5617
Negative predictive value	13.63%	21.42%	0.261	0.6094
Diagnostic accuracy	82.40%	85.00%	0.274	0.6006

Stratification by symptom duration in Table 7 demonstrated clinically relevant performance differences - specificity of 75.00% versus 50.00% and diagnostic accuracy of 84.52% versus 81.81% for presentations within versus beyond 48 hours, respectively - though neither difference reached statistical significance on chi-square testing (specificity: χ² = 0.686, p = 0.4077; accuracy: χ² = 0.312, p = 0.5765). These non-significant results are attributable to the critically small true-negative denominators (TN = 3 in each subgroup), substantially limiting the statistical power of intergroup specificity comparisons and warranting cautious interpretation of the observed clinical trends.

**Table 7:** Stratified Diagnostic Accuracy of Transvaginal Sonography with Respect to Duration of Symptomatology with Intergroup Comparison Data presented as absolute values and percentages. Symptom duration threshold set at 48 hours per study operational definition. P < 0.05 considered statistically significant.

Diagnostic Index	Symptom Duration ≤48 Hours (n=84)	Symptom Duration >48 Hours (n=121)	Chi-square value	p-value
True positive	68	96	-	-
False positive	1	3	-	-
False negative	12	19	-	-
True negative	3	3	-	-
Histopathology positive	80	115	-	-
Histopathology negative	4	6	-	-
Sensitivity	85.00%	83.47%	0.071	0.7898
Specificity	75.00%	50.00%	0.686	0.4077
Positive predictive value	98.55%	96.96%	0.408	0.5229
Negative predictive value	20.00%	13.63%	0.148	0.7003
Diagnostic accuracy	84.52%	81.81%	0.312	0.5765

The consolidated summary of diagnostic performance indices across all stratified subgroups established that transvaginal sonography maintained a consistently high positive predictive value ranging from 96.96% to 98.55% across all subgroups, reflecting the elevated disease prevalence inherent to this study population (Table 8). Sensitivity remained relatively stable across strata, ranging from 83.47% to 85.52%, demonstrating acceptable test performance regardless of age, gestational age, or symptom duration. Conversely, specificity exhibited the greatest variability across subgroups, ranging from 50.00% to 75.00%, with superior specificity demonstrated in participants with gestational age exceeding 27 weeks (75.00%) and those with symptom duration of 48 hours or less (75.00%), collectively identifying early presentation and advanced gestational age as the two most favourable clinical contexts for sonographic diagnostic deployment.

**Table 8:** Summary of Diagnostic Performance Indices of Transvaginal Sonography Across All Stratified Subgroups PPV = positive predictive value; NPV = negative predictive value. All values are expressed as percentages.

Subgroup	n	Sensitivity (%)	Specificity (%)	PPV (%)	NPV (%)	Accuracy (%)
Overall	205	84.10	60.00	97.62	16.21	82.93
Age 15-30 years	119	84.96	66.67	97.95	19.04	84.03
Age 31-49 years	86	84.14	50.00	97.18	13.33	82.55
Gestational age ≤27 weeks	125	84.03	50.00	97.08	13.63	82.40
Gestational age >27 weeks	80	85.52	75.00	98.48	21.42	85.00
Symptom duration ≤48 hours	84	85.00	75.00	98.55	20.00	84.52
Symptom duration >48 hours	121	83.47	50.00	96.96	13.63	81.81

## Discussion

The present cross-sectional study evaluated the diagnostic accuracy of TVS in detecting RPOC among 205 women of reproductive age, utilising histopathological examination as the reference gold standard. The overall findings demonstrated a sensitivity of 84.10%, specificity of 60.00%, positive predictive value (PPV) of 97.62%, negative predictive value (NPV) of 16.21%, and an overall diagnostic accuracy of 82.93%. These results collectively position TVS as a clinically valuable confirmatory diagnostic instrument, whilst simultaneously identifying its substantive limitations in disease exclusion - findings that resonate meaningfully with the broader published literature on sonographic diagnostic performance in RPOC and that carry direct implications for clinical pathway design in resource-constrained tertiary care settings.

The sensitivity of 84.10% reported in the present study demonstrates close concordance with findings reported by Iqbal et al. (2018), who conducted a diagnostic accuracy study of ultrasound in detecting RPOC at a tertiary care hospital in Karachi, Pakistan, among a comparable South Asian clinical population, reporting a sensitivity of 75.22% and specificity of 72.50% across their study cohort [8]. The present study's marginally superior sensitivity may reflect the methodological advantage of employing transvaginal Doppler sonography with a dedicated 5-7 MHz endovaginal probe, as opposed to transabdominal approaches, thereby enabling superior endometrial tissue characterisation and more precise delineation of intrauterine echogenic masses. Similarly, Matijevic et al. (2009), in their prospective evaluation of sonographic and clinical parameters for predicting RPOC, reported sensitivity values ranging from 79% to 93% depending upon the specific sonographic criterion applied, reinforcing that the choice of diagnostic threshold materially influences reported performance indices [9]. The present study's sensitivity of 84.10% falls comfortably within this established range, lending methodological credibility to the current findings.

Regarding modality selection, transvaginal sonography demonstrates established superiority over transabdominal sonography in RPOC diagnosis. Sundararajan et al. (2024), in their systematic review and meta-analysis evaluating ultrasound accuracy across multiple studies, demonstrated that an echogenic mass on TVS yielded a sensitivity of 91.5% (95% CI: 0.844-0.955), specificity of 84.3% (95% CI: 0.615-0.947), and diagnostic odds ratio of 57.787 - substantially superior to endometrial thickness measurement alone - directly supporting the methodological selection of TVS with colour Doppler in the present investigation over transabdominal approaches [13].

The specificity of 60.00% observed in the present investigation merits critical contextualisation against the broader literature. This value is notably lower than specificities reported in several contemporary studies, which have documented specificities ranging from 72% to 88% for grey-scale TVS combined with colour Doppler assessment. Atri et al. (2011), in their rigorous evaluation of the best predictors of grayscale ultrasound combined with colour Doppler for RPOC diagnosis among 53 patients, emphasised that demonstrable vascular flow patterns within echogenic intrauterine masses substantially improved specificity, with colour Doppler exhibiting superior discriminatory capacity compared to grey-scale imaging alone [10]. The relatively modest specificity in the present study may be partly attributable to the exceptionally high disease prevalence of 95.12% within the study cohort, which mathematically constrains the number of true negative cases available for specificity computation, thereby rendering this metric statistically vulnerable to imprecision in small denominators. Chopra et al. (2017), in their correlation study of ultrasound with histopathology for RPOC in medically managed abortions, similarly noted that high-prevalence clinical populations produced inflated PPV estimates and compressed specificity values - a methodological observation directly applicable to the present study's findings and one that necessitates cautious interpretation of specificity metrics in isolation from their epidemiological context [11].

The PPV of 97.62% documented in the present study, whilst clinically impressive on initial appraisal, must be interpreted with appropriate epidemiological caution. As comprehensively established by Sellmyer et al. (2013) in their radiographic and histological review of RPOC physiological and imaging features, PPV is a prevalence-dependent metric that demonstrates pronounced susceptibility to population-level disease frequency [5]. Given that 195 of 205 participants (95.12%) demonstrated histopathologically confirmed RPOC - a prevalence substantially exceeding general population estimates - the elevated PPV is mathematically expected rather than exclusively reflective of superior sonographic discriminatory performance. This observation is further reinforced by the seminal work of Sadan et al. (2004), who evaluated the role of sonography in RPOC diagnosis among 57 patients and demonstrated that while sonographic positive findings reliably predicted tissue retention, the standalone application of sonography without histopathological correlation carried an unacceptable rate of diagnostic misclassification, particularly in cases where endometrial echogenicity was ambiguous in the absence of demonstrable Doppler vascularity [14]. The NPV of 16.21% documented in the present study is the most clinically consequential finding, establishing unequivocally that a negative TVS result provides insufficient diagnostic reassurance for excluding RPOC in high-prevalence clinical settings - a conclusion that carries direct implications for patient triage and post-procedural surveillance protocols.

Convergent evidence from Wolman et al. (2009), who evaluated combined clinical and ultrasonographic workup for RPOC diagnosis in 102 women, further substantiated this position by demonstrating that multimodal diagnostic integration - encompassing clinical symptomatology, endometrial thickness measurement, and Doppler vascularity assessment - improved diagnostic confidence substantially compared to sonography alone, yet could not eliminate the need for histopathological confirmation in equivocal cases [15]. The present study's findings extend this evidence base by demonstrating that the diagnostic inadequacy of TVS in isolation is particularly pronounced among patients with sub-acute or prolonged symptom duration - a subgroup in whom sonomorphological ambiguity is mechanistically expected due to progressive tissue degradation and secondary endometrial inflammatory change.

Beyond conventional diagnostic indices, likelihood ratio analysis provides prevalence-independent metrics of TVS discriminatory capacity. The positive likelihood ratio (LR+ = 2.10) in the present study indicates only a modest upward probability revision following a positive TVS result. Matijevic et al. (2009), in their prospective cohort of 93 patients, specifically reported that the likelihood ratio of sonography alone was 1.47 (95% CI: 1.25-1.84), improving to 2.16 (95% CI: 1.30-3.59) with colour Doppler augmentation - findings directly concordant with the present study. The negative likelihood ratio of 0.27, substantially exceeding the 0.10 exclusionary threshold, confirms TVS's limitation as a standalone rule-out instrument [9].

The age-stratified analysis revealed that younger women (15-30 years) demonstrated marginally superior diagnostic accuracy (84.03%) compared to older women (31-49 years, 82.55%), with a more pronounced divergence in specificity (66.67% versus 50.00%, respectively). This age-dependent variation in sonographic diagnostic performance has not been extensively characterised in the published literature, representing a contributory analytical finding of the present investigation. Ustunyurt et al. (2008), in their dedicated analysis of TVS in RPOC diagnosis among 73 women, acknowledged that uterine structural heterogeneity associated with advancing reproductive age - including coexistent adenomyosis, intramural fibroids, and endometrial polyps - could introduce sonomorphological confounders that reduce specificity by mimicking or obscuring the echogenic appearance of retained trophoblastic tissue [7]. This mechanistic explanation is directly consistent with the present study's age-stratified performance gradient and suggests that supplementary sonographic evaluation modalities, such as sonohysterography, may offer particular diagnostic value in older reproductive-age women where anatomical confounders are more prevalent.

Gestational age-stratified analysis demonstrated that TVS performance was demonstrably superior in participants with gestational age exceeding 27 weeks, achieving sensitivity of 85.52%, specificity of 75.00%, and diagnostic accuracy of 85.00%, compared to 84.03%, 50.00%, and 82.40%, respectively, in the ≤27-week subgroup. These findings are robustly corroborated by Esmaeillou et al. (2015), who demonstrated in their prospective study of 86 patients that colour Doppler sonography exhibited significantly enhanced diagnostic accuracy for RPOC detection following second-trimester abortion compared to first-trimester cases, attributing this differential to the greater trophoblastic tissue volume and more pronounced intratissue vascular signals present at advanced gestational ages, which facilitate definitive sonographic characterisation [6]. Durfee et al. (2005), in their comprehensive characterisation of sonographic and colour Doppler features of RPOC among 41 patients, similarly established that echogenic intrauterine masses with demonstrable colour Doppler vascularity were the most reliable sonographic predictors of RPOC, with intramass vascularity being more consistently demonstrable at advanced gestational ages - a finding directly supportive of the gestational age-related performance gradient observed in the present study [16]. Furthermore, Achiron et al. (1993) demonstrated in their transvaginal duplex Doppler ultrasonography study among bleeding patients suspected of harbouring residual trophoblastic tissue that Doppler vascularity indices were significantly more robust and diagnostically discriminatory in cases involving larger retained tissue volumes, consistent with the superior specificity observed in the advanced gestational age subgroup of the present investigation [17].

The stratification by symptom duration yielded one of the most clinically actionable findings of the present investigation. Participants presenting within 48 hours of symptom onset demonstrated superior specificity (75.00%) and diagnostic accuracy (84.52%) compared to those presenting beyond 48 hours (50.00% and 81.81%, respectively). This temporal performance gradient carries substantive implications for the design of clinical diagnostic pathways and patient referral protocols. Abbasi et al. (2008), in their evaluation of clinical and ultrasound findings in RPOC diagnosis among 96 women, established that delayed presentation was associated with progressive tissue degradation, secondary endometrial inflammatory changes, and increasing sonomorphological complexity - all of which introduce diagnostic ambiguity and systematically reduce specificity by generating false-positive echogenic signals from inflammatory debris [18]. Furthermore, Mulic-Lutvica and Axelsson (2006), in their prospective cohort study of 61 women with secondary postpartum haemorrhage, demonstrated that echogenic intrauterine masses identified on ultrasound were strongly associated with retained placental tissue, but crucially acknowledged that the sonographic appearance of RPOC evolved significantly with time from the index obstetric event, with older retained tissue demonstrating reduced echogenicity and diminished Doppler vascularity - both of which contribute to the declining specificity observed in the present study's sub-acute presentation subgroup [19].

The global burden of RPOC must also be appreciated within its broader epidemiological context to fully understand the public health significance of accurate diagnostic stratification. Adler et al. (2012), in their systematic quantification of the global burden of morbidity attributable to unsafe abortion, established that incomplete evacuation of uterine contents and consequent RPOC represent a disproportionately large contributor to maternal morbidity in low- and middle-income countries, with hospital-based studies consistently underestimating true incidence due to structural diagnostic inadequacies [3]. Sathar et al. (2014) further contextualised this burden within the Pakistani clinical landscape, demonstrating that induced abortions and unintended pregnancies in Pakistan generate a substantial reservoir of RPOC cases presenting to tertiary care facilities - a demographic reality directly reflected in the exceptionally high disease prevalence of 95.12% observed in the present study's cohort from BMCH, Quetta [4]. The regional epidemiological context established by these foundational studies underscores the particular relevance and translational significance of the present investigation's findings for clinical practice in Pakistan and comparable South Asian settings.

The overall diagnostic accuracy of 82.93% reported in the present study compares favourably with findings from Karimpour (2010), who documented a diagnostic accuracy of approximately 80% for transvaginal sonography in correlation with pathological findings and clinical examination among a comparable Middle Eastern population [12]. Zare and Zijerdi (2010) similarly reported that ultrasound demonstrated reasonable but imperfect predictive capacity for RPOC following first-trimester spontaneous abortion, with accuracy estimates in the low-to-mid 80% range - directly concordant with the present study's overall performance and reinforcing the cross-regional consistency of TVS diagnostic limitations [20]. Shannon et al. (2004), in their systematic literature review of infection following medical abortion, further emphasised that delayed or missed RPOC diagnosis - a direct consequence of sonographic false-negative findings - significantly elevated the risk of ascending uterine infection, endometritis, and sepsis, thereby establishing a direct mechanistic link between diagnostic accuracy limitations and clinically adverse patient outcomes [21]. This evidence strengthens the present study's argument for mandatory histopathological corroboration in all sonographically negative but clinically symptomatic cases.

The morbidity associated with untreated or misdiagnosed RPOC extends beyond acute infectious complications. Hanstede et al. (2015), in their centralised analysis of 232 Asherman's syndrome cases managed surgically between 2003 and 2013, identified retained products of conception and their surgical management as a prominent antecedent of intrauterine adhesion formation, with the risk of adhesion development being particularly pronounced following repeated uterine instrumentation performed on the basis of erroneous or unconfirmed diagnoses [2]. Conversely, Smorgick et al. (2014), in their meta-analysis and literature review of hysteroscopic management of RPOC encompassing 609 cases, demonstrated that accurate pre-operative sonographic identification of RPOC enabled targeted hysteroscopic removal with significantly lower adhesion formation rates compared to blind curettage - reinforcing that sonographic diagnostic precision, even when imperfect, carries meaningful downstream therapeutic consequences [22]. Barel et al. (2015) extended this analysis by identifying specific risk factors for intrauterine adhesion formation following hysteroscopic RPOC treatment, establishing that the extent of retained tissue - which is sonographically estimable - constituted an independent predictor of post-procedural adhesive disease, further underscoring the clinical value of optimised sonographic characterisation within a histopathologically anchored diagnostic framework [23].

Taken collectively, the present study's findings establish that transvaginal sonography functions most effectively as a confirmatory diagnostic modality for RPOC - particularly in younger women, at advanced gestational ages, and during the acute symptomatic phase within 48 hours of onset. The high PPV strongly supports its utility in triaging patients for surgical or medical intervention, while the critically low NPV necessitates histopathological corroboration in all clinically equivocal cases. These conclusions are consistent with the emerging multimodal diagnostic consensus articulated across the contemporary literature, which advocates for the systematic integration of TVS within a broader clinical and pathological framework rather than its deployment as a standalone diagnostic arbiter.

Clinical significance

The findings of this study carry direct and substantive clinical implications for the management of women presenting with suspected RPOC in resource-constrained tertiary care settings. Transvaginal sonography, given its non-invasive nature, immediate availability, and acceptable sensitivity of 84.10%, should be integrated as the primary first-line investigative modality in the diagnostic workup of suspected RPOC. The exceptionally high PPV of 97.62% establishes that a positive TVS result reliably justifies proceeding to surgical evacuation, thereby supporting clinical decision-making efficiency. However, the critically low NPV of 16.21% mandates that a negative sonographic result must not be accepted as definitive exclusion of RPOC, particularly in high-prevalence clinical environments. Clinicians should maintain a low threshold for proceeding to histopathological confirmation in symptomatic women with negative TVS findings, especially those presenting beyond 48 hours of symptom onset or at earlier gestational ages, where sonographic specificity is demonstrably compromised.

Strengths of the study

The present study demonstrates several methodological strengths that enhance its scientific credibility. The use of histopathology as the reference gold standard ensures diagnostic certainty and minimises verification bias. The adequately powered sample size of 205, calculated using a validated formula, provides sufficient statistical precision for diagnostic index estimation. The systematic stratification of diagnostic performance across age, gestational age, and symptom duration represents a distinctive analytical contribution that extends beyond the scope of many comparable regional studies. Furthermore, the consecutive non-probability sampling technique ensured representative enrolment of all eligible participants, minimising selection bias. The utilisation of colour Doppler transvaginal sonography, rather than grey-scale imaging alone, represents a methodological strength that enabled more comprehensive vascular characterisation of intrauterine contents.

Limitations

Several limitations warrant acknowledgment in interpreting the present findings. The exceptionally high disease prevalence of 95.12% within the study cohort substantially constrains the reliability of specificity and NPV estimates, as only 10 histopathologically negative cases were available for analysis - a sample insufficient for robust specificity computation. The single-centre design at BMCH, Quetta limits the external generalisability of findings to other institutional and geographic contexts. The study did not systematically document specific sonographic criteria applied during TVS evaluation - such as endometrial thickness thresholds or Doppler vascularity scoring - which precludes subgroup analysis by sonographic criterion. Additionally, operator-dependent variability in TVS performance was not formally assessed or controlled, representing a potential source of diagnostic heterogeneity. Finally, the cross-sectional design precludes causal inference regarding the temporal relationship between clinical variables and diagnostic performance.

Recommendations

Future multicentre diagnostic accuracy studies with balanced disease prevalence and standardised TVS reporting criteria - incorporating defined endometrial thickness thresholds and colour Doppler vascularity grading - are recommended to generate more robust specificity and NPV estimates. Integration of sonohysterography and three-dimensional TVS modalities should be explored to augment diagnostic precision. Clinical protocols mandating histopathological confirmation in TVS-negative symptomatic cases should be institutionally implemented.

## Conclusions

The present study establishes that transvaginal sonography demonstrates acceptable sensitivity and overall diagnostic accuracy for the detection of retained products of conception, with a sensitivity of 84.10%, PPV of 97.62%, and diagnostic accuracy of 82.93% against histopathological gold standard. Diagnostic performance was superior in younger women, at gestational ages exceeding 27 weeks, and among patients presenting within 48 hours of symptom onset. However, the critically low NPV of 16.21% and modest specificity of 60.00% confirm that TVS cannot function as a standalone exclusionary diagnostic tool in high-prevalence clinical populations. Histopathological confirmation remains indispensable in all sonographically equivocal or negative cases presenting with persistent clinical symptoms. Transvaginal sonography should therefore be systematically deployed as a confirmatory first-line modality within a multimodal diagnostic framework that mandates histopathological corroboration to minimise diagnostic misclassification and its associated clinical sequelae such as haemorrhage, sepsis, intrauterine adhesive disease.
